# Tau pathology differs by sex in Alzheimer's disease in Down syndrome

**DOI:** 10.1002/alz.70838

**Published:** 2025-10-23

**Authors:** Xu‐Qiao Chen, Xinxin Zuo, William C. Mobley

**Affiliations:** ^1^ Department of Neurosciences School of Medicine University of California San Diego La Jolla California USA

**Keywords:** Alzheimer's disease, *APP*, Down syndrome, PHF1 tau, sarkosyl‐insoluble, tau

## Abstract

**INTRODUCTION:**

Alzheimer's disease (AD), the leading cause of dementia, is more common in females. Although sex differences in tau pathology have been reported in AD, findings remain inconsistent. Down syndrome (DS), caused by trisomy 21, is the most common genetic cause of AD (DS‐AD) and features tau pathology, but sex effects in DS‐AD remain unclear.

**METHODS:**

We examined *post mortem* brain samples from individuals with DS‐AD, DS without AD, and a rare partial trisomy 21 (PT) case with only two amyloid precursor protein (*APP*) gene copies. PHF1 tau, total tau, and sarkosyl‐soluble and insoluble fractions were quantified by group and sex.

**RESULTS:**

PHF1 tau was significantly elevated in DS‐AD, especially in females. Lower total tau in DS‐AD males explained the absence of sex differences after normalization. Sarkosyl‐insoluble tau was also higher in DS‐AD females. DS without AD, and the PT case showed minimal pathology.

**DISCUSSION:**

These findings suggest sex‐specific tau dynamics in DS‐AD and support a role for *APP* dosage.

**Highlights:**

- Tau pathology is significantly elevated in individuals with DS‐AD, especially in females.
- Female DS‐AD brains show markedly higher PHF1 (S396/404) and sarkosyl‐insoluble tau levels compared to males.
- The observed sex difference in phosphorylated tau is driven by lower total tau in DS‐AD males.
- Minimal tau pathology is present in DS without AD and in a rare partial trisomy 21 case.
- These findings implicate *APP* gene dosage in tau pathology in DS‐AD.

## 1 BACKGROUND

Alzheimer's disease (AD), the predominant cause of dementia, accounts for approximately 70% of cases. It is also among the leading causes of mortality.^1^ The clinical presentation includes memory loss, cognitive decline, behavioral disturbances, and difficulties performing daily activities. Many studies document a higher prevalence of AD in women compared to men.^1^ Research also suggests potential variations between males and females in the progression of tau pathology in AD, with some reporting more rapid tau accumulation in females as measured using positron emission tomography (PET) scanning.^2^, ^3^ Emerging evidence also suggests that women with AD tend to exhibit elevated levels of tau protein in cerebrospinal fluid (CSF) and brain.^4^, ^5^ However, not all studies support sex‐related disparities in tau pathology,^6^ suggesting that differences in sample size, participant characteristics, and methodological approaches may be responsible for discrepant findings. Given the significance of tau pathology in disease, further investigation is needed to document the extent to which sex differences exist in tau pathology and, if present, to decipher the underlying mechanisms, define the implications for disease progression, and discover effective treatments.

Down syndrome (DS), the leading genetic cause of AD (DS‐AD),^7^ results from an extra copy of chromosome 21 (HSA21). During early life, DS presents with systemic changes impacting the heart and other tissues, distinctive facial features, developmental delay, and intellectual disabilities; in late life, most individuals show the pathological and clinical features of AD.^8^, ^9^ An increase in the dose of the amyloid precursor protein (*APP*) gene, located on HSA21, is necessary for DS‐AD.^9^ Some reports point to sex differences for the pathological manifestations of AD in DS.^10^ Highlighting a potential sex‐related influence on tau pathology, females with DS often present with elevated levels of neurofibrillary tangles (NFTs).^11^ However, whether or not females and males with DS differ in tau pathology requires further study to assess the levels of total tau and phosphorylated tau. To explore possible sex differences, herein we measured the levels of PHF1 tau, a well‐studied phosphorylated isoform of tau (S396/404), and total tau in RIPA extracts, as well as in sarkosyl‐soluble and insoluble extracts. Our studies included people with DS‐AD, DS without AD, a rare case of partial trisomy 21 (PT) whose genome harbored only two copies of *APP*,^12^ and controls.

## 2 METHODS

### 2.1 Ethics approval

All procedures related to human samples were carried out under a protocol reviewed and approved by the University of California San Diego's Human Subjects Review Board (Institutional Review Board No.: 180620).

### 2.2 *Post mortem* human sample

Frontal cortex samples from individuals with DS, DS‐AD, and control groups were obtained from the National Institutes of Health (NIH) NeuroBioBank and the University of California Irvine Alzheimer's Disease Research Center (UCI ADRC) (Tables S1–S3).

### 2.3 SDS‐PAGE immunoblotting

Published protocols were followed for SDS‐PAGE immunoblotting^13^.

**RESEARCH IN CONTEXT**

1. **Systematic review**: Sex differences in AD have been reported in clinical progression and tau pathology, but findings are inconsistent. In DS‐AD, limited studies have addressed sex‐specific differences in tau accumulation, and biochemical comparisons across sexes are scarce.
2. **Interpretation**: Our study demonstrates that females with DS‐AD exhibit greater PHF1‐positive tau and aggregated sarkosyl‐insoluble tau than males. These differences are not explained by tau gene expression, suggesting sex‐specific post‐translational regulation of tau. The absence of pathology in the partial trisomy 21 case supports a key role for *APP* gene dosage in tau accumulation.
3. **Future directions**: Future research should explore mechanisms underlying sex differences in tau aggregation, such as hormonal regulation or protein clearance pathways, and assess their relevance to therapeutic development. Larger, sex‐balanced studies in DS‐AD and sporadic AD may help guide precision medicine strategies.

Detailed methods are described in the Supplementary Information.

## 3 RESULTS

### 3.1 Elevated PHF1 tau in DS‐AD and sex‐dependent differences

**FIGURE 1 alz70838-fig-0001:**
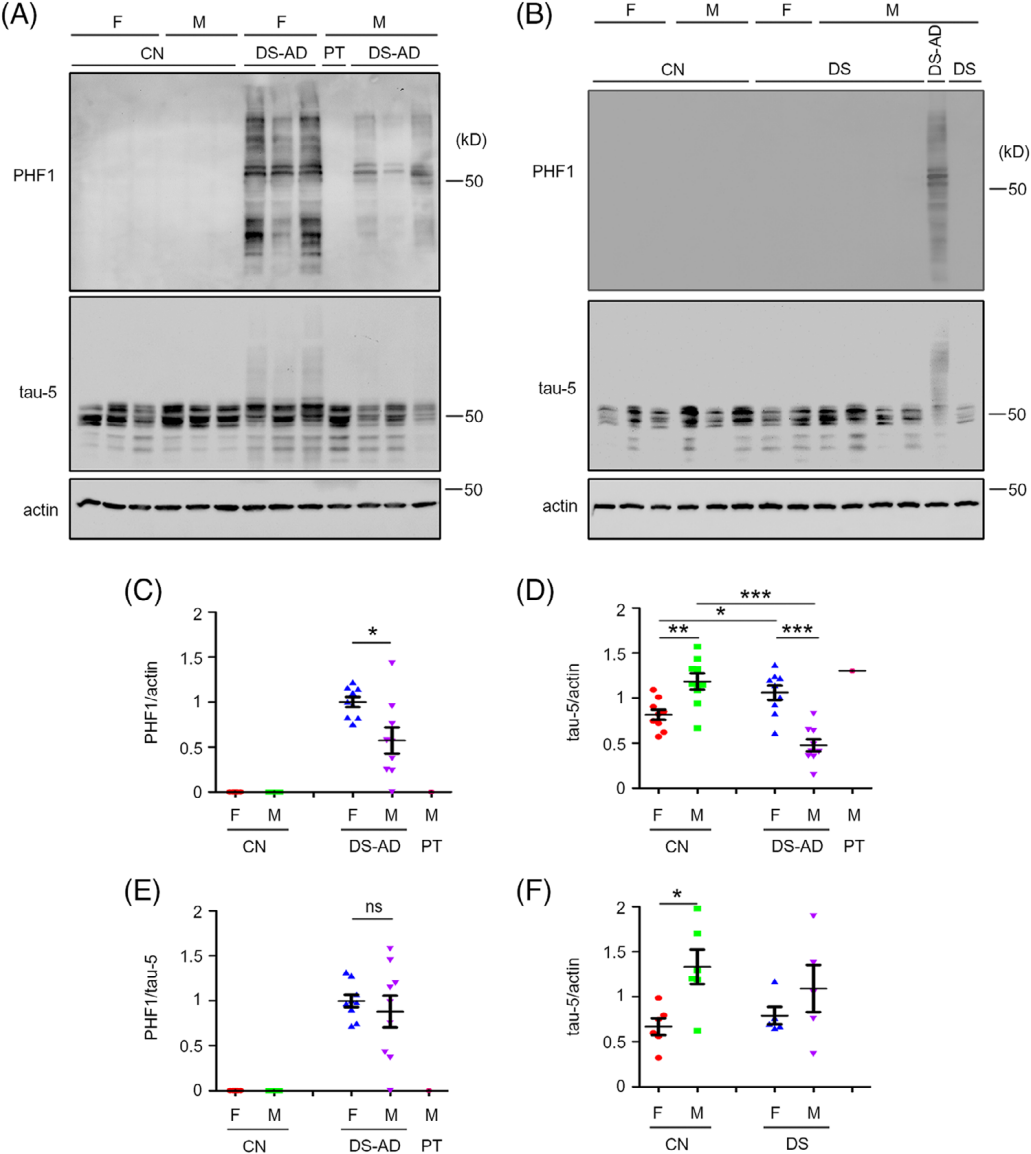
Sex differences in total tau and PHF1 tau levels in frontal cortex lysates of DS‐AD and DS. (A) Western blotting of PHF1 tau and total tau (tau‐5) in RIPA extracts from frontal cortex of subjects with DS‐AD and PT‐DS. β‐Actin was used as a loading control. (B) Western blotting of PHF1 tau and total tau in frontal cortex of subjects with DS. β‐Actin was used as loading control. (C) Quantitation and statistical analysis of the levels of PHF1 tau normalized to actin from DS‐AD and PT‐DS samples. (D) Quantitation and statistical analysis of total tau levels normalized to actin from DS‐AD and PT‐DS samples. (E) Quantitation and statistical analysis of the levels of PHF1 tau normalized to total tau from DS‐AD and PT‐DS samples. (F) Quantitation and statistical analysis of total tau levels normalized to actin from DS samples. The male DS‐AD case in panel (B) was from the samples in panel (C) and served as a positive control for PHF1 tau. F = female; M = male; CN = cognitively normal controls. DS‐AD, *n* = 18 (F: 9; M: 9); DS, *n* = 10 (F: 5; M: 5); and control for DS‐AD, *n* = 18 (F: 9; M: 9); control for DS, *n* = 12 (F: 6; M: 6). For panels (C) and (E), the average value of female DS‐AD was set to 1 in each group, whereas for panels (D) and (F), the average value of CN (female and male combined) was set to 1. One‐way ANOVA test followed by Newman–Keuls multiple comparison tests for panel (D); Mann–Whitney test for other panels: *p < 0.05, **p < 0.01, ***p < 0.001. AD, Alzheimer's disease; Down syndrome, PT, partial trisomy 21.

PHF1 tau was barely detected in RIPA extracts from cognitively normal control subjects but was readily detected in the frontal cortex of individuals with DS‐AD. Immunoblots revealed bands at ∼50 to 60 kD (tau monomers), as well as faster‐ and slower‐migrating species, the latter consistent with the PHF1 tau in higher‐molecular‐weight complexes (Figures 1A and S1A). Significantly, PHF1 tau was absent in DS without AD (Figures 1B and S1B), consistent with the increase in tau hyperphosphorylation in the DS‐AD but not the DS brain.^14^ Notably, females with DS‐AD showed markedly higher PHF1 levels than males (Figure 1A,C). The failure to detect PHF1 in the PT case (Figure  1A,C) is consistent with the absence of AD neuropathology at autopsy^12^ and with evidence in DS,^12^, ^15^ as well as in the Dp16 animal model,^14^ which links increased *APP* gene dosage to AD.

To explore whether sex differences in tau phosphorylation reflected differences in total tau, we measured total tau levels in RIPA lysates from DS, DS‐AD, and controls. Interestingly, male controls had slightly but significantly higher total tau levels than female controls (Figure 1A,D). Compared to controls, females with DS‐AD exhibited a ∼40% increase in total tau levels, whereas males with DS‐AD showed a ∼60% decrease. Within the DS‐AD group, males thus showed significantly lower total tau levels than females (Figure 1D). When PHF1 tau was normalized to total tau, the sex difference was no longer evident, suggesting that differences in total tau underlay the PHF1 difference (Figure 1E). In contrast, total tau levels in DS cases without AD did not differ significantly from controls for either sex (Figure 1F). The male PT case exhibited total tau levels comparable to male controls (Figure 1A,D). The data for the PT case are evidence that increased *APP* gene dose contributes both to elevated PHF1 tau and reduced total tau in males with DS‐AD.

### 3.2 Increased sarkosyl‐insoluble tau in females with DS‐AD

**FIGURE 2:**
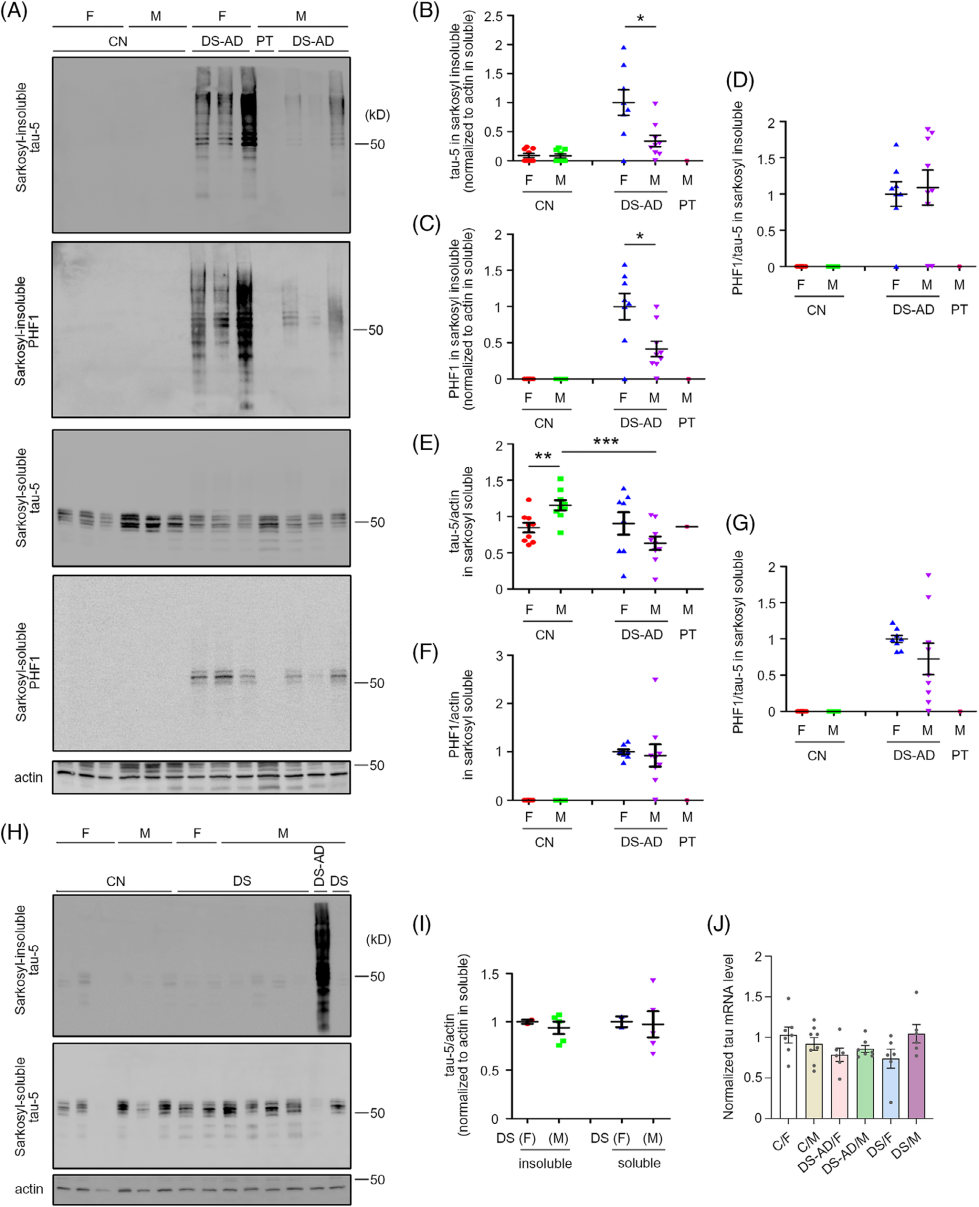
Sex differences in total tau and PHF1 tau levels in sarkosyl‐insoluble and ‐soluble fractions of the frontal cortex in DS‐AD and DS. (A) Western blot analysis of total tau and PHF1 tau in sarkosyl‐soluble and insoluble fractions from frontal cortex extracts of DS‐AD subjects. (B and C) Quantitation and statistical analysis of levels of total tau (B) and PHF1 tau (C) in sarkosyl‐insoluble fractions normalized to actin in sarkosyl‐soluble fractions from DS‐AD and PT‐DS samples. (D) Quantitation and statistical analysis of PHF1 tau/total tau ratio in sarkosyl‐insoluble fractions from DS‐AD. (E and F) Quantitation and statistical analysis of levels of total tau (E) and PHF1 tau (F) in sarkosyl‐soluble fractions normalized to actin in sarkosyl‐soluble fractions from DS‐AD and PT‐DS samples. (G) Quantitation and statistical analysis of PHF1 tau/total tau ratio in sarkosyl‐soluble fractions from DS‐AD. (H) Western blot analysis of total tau and PHF1 tau in sarkosyl‐soluble and insoluble fractions from frontal cortex extracts of DS subjects. (I) Quantification and statistical analysis of total tau levels in sarkosyl‐insoluble and soluble fractions normalized to actin in sarkosyl‐soluble fraction from DS samples. The male DS‐AD case shown in panel (H) was selected from the samples in panel (B) and served as a positive control for total tau, with a longer exposure used to reveal potential signals in other lanes. DS‐AD, *n* = 17 (F: 9; M: 8); DS, *n* = 7 (F: 2; M: 5); and control for DS‐AD, *n* = 18 (F: 9; M: 9); control for DS, *n* = 6 (F: 3; M: 3). (J) Relative tau mRNA levels in frontal cortex of DS‐AD, DS, and control groups were quantified by qPCR, using *ACTB* mRNA as internal reference. For panels (B–D), (F), and (G), the average value of female DS‐AD was set to 1 in each group, whereas for panel (E), the average value of CN (female and male combined) was set to 1. For panel (I), the average value of female DS in both the insoluble and soluble fractions was set to 1, while in panel (J), the average value of female CN was set to 1. *n* = 6 to 8. Mann–Whitney test: *p < 0.05, **p < 0.01, ***p < 0.001. AD, Alzheimer's disease; Down syndrome, PT, partial trisomy 21.

Aggregated proteins, such as amyloid beta (Aβ) and phosphorylated tau, are typically enriched in the sarkosyl‐insoluble fraction because they resist solubilization.^16^ Given the high‐molecular‐weight PHF1 tau complexes in RIPA lysates from DS‐AD brains, we asked if tau isoforms in sarkosyl‐soluble and ‐insoluble fractions differed by sex. To assess this, we prepared both fractions and analyzed total tau and PHF1 tau in the DS‐AD, DS, and control frontal cortex. As expected, little if any total tau was detected in the sarkosyl‐insoluble fractions from control brains. In contrast, DS‐AD samples displayed abundant high‐molecular‐weight tau species (Figures 2A,B and S2). Controls and DS‐AD samples also differed significantly when sarkosyl‐insoluble samples were probed with the PHF1 antibody (Figures 2A,C and S2). Thus, aggregates of total tau and PHF1 tau were detected in the DS‐AD but not the control brain. As for the RIPA lysates, total tau and PHF1 tau levels were higher in females than males and the PHF1/total tau ratio showed no sex difference (Figure 2A,D). In the sarkosyl‐soluble fraction of DS‐AD samples, total tau mainly migrated at the monomer weight; the levels in males were lower than in females, but the difference was not significant (Figures 2A,E and S2). PHF1 tau signals appeared in the sarkosyl‐soluble fractions of DS‐AD samples, but not in controls. Statistical analysis demonstrated no significant sex difference in PHF1 tau levels or the PHF1/total tau ratio in the sarkosyl‐soluble fractions of DS‐AD (Figures 2A,F,G and S2).

Sarkosyl‐insoluble fractions from DS and control brains demonstrated minimal total tau, primarily migrating at the monomer size (∼50 to 60 kD), while sarkosyl‐soluble total tau was readily detected in DS brains (Figure 2H). There was no sex difference in total tau levels in both the insoluble and soluble fractions in DS cases without AD (Figure 2H,I). In the PT case, no tau was detected in the sarkosyl‐insoluble fraction, and PHF1 tau was absent from both soluble and insoluble fractions (Figure 2A–C,F), further supporting a role for increased *APP* gene dose in DS‐AD tau pathology.

*Post mortem* interval (PMI) could influence protein stability, but no sex‐related PMI differences were found within DS‐AD or control groups (Table S4). Across sex‐dependent measures, PMI correlated only with total tau in the sarkosyl‐soluble fraction of male DS‐AD brains (Table S5), suggesting possible sex‐specific sensitivity of soluble monomeric tau to PMI in male DS‐AD, although this requires caution due to limited sample size. No age differences were observed between sexes in DS‐AD or controls, but within the DS group, males were younger than females (Table S4). In addition, age showed no associations with total tau or PHF1 tau across groups, except in control for DS‐AD (C/DS‐AD) females, where it correlated positively with total tau in whole lysates and sarkosyl‐soluble fractions (Table S5).[Fig alz70838-fig-0001]

### 3.3 Tau mRNA expression is unchanged across sex and disease status

To determine whether observed changes in protein levels might reflect differences in tau transcription, we quantified tau mRNA levels across control, DS‐AD, and DS groups. Tau mRNA levels did not differ between sexes in the control, DS‐AD, or DS groups, nor between the control and either the DS‐AD or DS groups (Figure 2J). These findings reinforce the evidence that the accumulation of high‐molecular‐weight tau in sarkosyl‐insoluble fractions is a pathological feature of DS‐AD. These data for DS‐AD mirror those in AD.^17^

## 4 DISCUSSION

Sex differences in AD have been reported across prevalence, onset, imaging, biomarkers, and neuropathology, though findings remain inconsistent.^18^ Women show higher prevalence and faster progression than men.^18^ Aβ‐positive females demonstrate more rapid accumulation of NFTs than males.^19^ Additionally, females with AD show higher NFT densities than males.^20^ In DS, some studies report earlier onset and increased post‐menopausal dementia risk in women, while others find no significant sex differences.^6^, ^10^, ^21^ Neuropathologically, DS‐AD women also have greater NFT densities.^11^ Inconsistencies may stem from variable sample sizes, methods, and limited sex‐specific reporting, underscoring the need for further research. These differences suggest that hormonal, genetic, and immune‐related mechanisms may influence AD pathogenesis and progression in both the neurotypical and DS populations. Given the clinical importance of tau pathology, sex‐specific variations in tau accumulation, post‐translational modifications, and regional distributions may serve to guide studies of pathological mechanisms and inform the development of targeted therapeutic strategies.

We found that PHF1 immunoreactivity was present exclusively in DS‐AD samples, not in controls, DS individuals without AD, or the PT case. PHF1 levels were particularly increased in both high‐ and low‐molecular‐weight species detected in whole‐cell RIPA lysates and sarkosyl‐insoluble fractions, while only low levels of monomer‐sized PHF1 were observed in the sarkosyl‐soluble fraction. The sex difference in DS‐AD for PHF1 tau levels paralleled differences in total tau levels. In sarkosyl‐insoluble fractions, relative to controls, DS‐AD cases showed increases in both tau and PHF1 levels; the increases in females were greater than in males. Interestingly, the relative sex differences in sarkosyl‐insoluble lysates were also greater than in RIPA lysates (female to male: total tau/actin [RIPA = 2.1; sarkosyl‐insoluble to soluble = 2.9]; PHF1 [RIPA = 1.75; sarkosyl‐insoluble = 2.4]). We are unaware of other studies that examined tau pathology in women and men at the biochemical level, as we have. However, a recent study using quantitative immunostaining reported greater tau pathology in the frontal and occipital cortices of women with DS‐AD than in men, reflected by a trend toward higher AT8 labeling. No sex difference was observed for Aβ pathology. The paper suggests that women may experience faster progression of AD‐related neuropathology than men, though possibly not in the frontal cortex.^22^ Our study, employing a different method to examine tau, reveals a much greater difference between women and men, suggesting that biochemical methods might be more sensitive in detecting pathological tau species.

Our findings indicate increases in DS‐AD females in both total tau and PHF1 tau without corresponding changes in tau mRNA, suggesting a post‐transcriptional mechanism. Several points emerge. First, male controls have more total tau than female controls. Second, DS males and females have total tau levels that do not differ from their controls, albeit with a trend for an increase in males that approximates the increase in male controls. Third, the differences of interest are two‐fold: Males show a decrease in total tau relative to controls, while females show an increase, resulting in a significant sex difference. There are two possible explanations for the increase in total tau in females. One is that in females, some event(s) increase susceptibility to tau phosphorylation and its aggregation, thereby reducing turnover of total tau. We envision the possibility that this could be driven by estrogen‐related modulation of tau via differences in the activity of tau‐directed kinases and phosphatases.^23^ Interestingly, earlier menopause is strongly associated with increased risk of earlier onset of AD in women with DS.^24^ Whether menopause is linked to sex‐dependent tau pathology needs to be addressed in future studies. An alternative explanation is that males may be more effective at clearing pathological forms of tau through autophagic or proteosomal mechanisms. Future studies will be required to answer this question, possibly including studies in male and female tauopathy models. Interesting, but as yet unexplained, is that greater pathology in females is not reflected in an increase in the age‐related prevalence of symptomatic AD as compared to males.^25^

Considering the close linkage between tau pathology and cognitive decline in AD,^26^ unraveling the mechanisms that underlie sex differences may reveal novel therapeutic targets and guide personalized treatments for DS‐AD. In a prior study, we noted significantly higher APP C‐terminal fragment (APP‐CTF) levels in females with DS‐AD and DS,^14^ suggesting sex differences in APP metabolism. The effect was greater than could be accounted for by *APP* gene dose, as reflected in the levels of full‐length APP, suggesting the contribution of other factors and supported by emerging evidence that sex hormones like estrogen and testosterone, immune responses, and genetic risk profiles may contribute to sex‐specific vulnerability in tau pathology and neurodegeneration.^10^, ^23^ Our earlier research demonstrated that in DS‐AD, increased *APP* gene dosage resulted in comparably elevated APP and Aβ levels in males and females relative to control brains.^14^, ^27^ Therefore, there is currently no evidence that APP or Aβ levels explain the sex differences in tau. The recent paper mentioned earlier found no difference in Aβ immunostaining between females and males with DS‐AD.^22^ Increasing evidence indicates that the β‐CTF processing product of APP promotes hyperactivation of the small GTPase Rab5,^28^, ^29^ which regulates the formation, trafficking, and function of early endosomes.^30^ Using antisense oligonucleotides targeting Rab5 mRNAs to normalize Rab5 activation in the Dp16 mouse model reduced the levels of tau phosphorylation to those found in euploid controls, implicating Rab5 activation status in tau hyperphosphorylation.^31^ Thus, in females, increased β‐CTF may influence tau pathology through Rab5 in DS.

Regarding the link between tau pathology and neuronal loss in DS, especially concerning sex‐related differences, there are limited data available. However, Weigel et al.^32^ reported that, relative to adults aged 26 to 41 – the end of the developmental period – neuron loss first appears in the 43 to 49 age group, averaging ∼12% and showing regional variability with statistical significance only in the caudate nucleus. In contrast, individuals aged 49 to 59 exhibit significant neuronal loss across multiple regions, including the entorhinal cortex, hippocampal CA fields, subiculum, and amygdala. Data for the frontal cortex and sex‐specific differences were not provided. Nonetheless, most cortical neuron loss appears to occur during the 50s, coinciding with the transition from mild to moderate and severe dementia, as well as Braak stage VI and Thal phase 5 pathology. Future studies are needed to better understand the relationship between neuronal loss and tau pathology in DS.

These findings build on our previous work examining a different phosphorylated tau species, pT212, in DS‐AD, DS, and control cohorts.^14^ Using comparable RIPA extracts, we found increased pT212 levels in both males and females with DS‐AD, but not in DS females or controls. Although females were not directly compared to males, we noted that, relative to actin, the pT212 levels in females were only ∼ 50% higher than in males. Studies of total tau in the same extracts confirmed that, relative to actin, the levels in females were higher than in males (∼50%). Thus, the sex difference in PHF1 tau demonstrated herein was greater than for pT212 in DS‐AD, suggesting that phosphorylated tau epitopes differ in their response to progression from DS to DS‐AD. It is perhaps relevant that residue 212 in tau is a substrate for DYRK1A, a gene on HSA21. pT212 may represent an early phosphorylation event in DS‐AD.^33^ Interestingly, pT212 levels were significantly lower in sporadic AD than in DS‐AD cases.^14^ The absence of differences relative to controls for pT212 and for tau levels in the PT case further supports a role for increased *APP* gene dose in tau pathology in DS.

In the supplementary figures, Group 3 samples include only female subjects from the University of California Irvine, to balance the DS‐AD case numbers across sexes. They were analyzed separately, which may limit the generalizability of the findings. Larger studies are needed to confirm these findings.

In summary, the observed sex differences in tau aggregation in DS‐AD underscore the need to explore genetic, hormonal, and molecular interactions in AD pathogenesis. These insights may guide sex‐specific diagnostic and therapeutic strategies in both DS and the neurotypical population. Limitations include a small sample size, potential bias from unmeasured factors such as hormonal status or comorbidities, and restriction to a single brain region. Larger, longitudinal studies are needed to validate and extend these findings.

## CONFLICT OF INTEREST STATEMENT

W.C.M. serves as a scientific advisory board (SAB) member and holds stock options from Alzheon, Inc. and Promis, Inc. W.C.M. also serves as a SAB member and holds stock in Acta Pharmaceuticals, Inc. His name is on a patent under the University of California San Diego, and Massachusetts General Hospital concerning γ‐secretase modulators licensed to Acta Pharmaceuticals, Inc. He has served as a consultant to AC Immune. W.C.M. holds a leadership position in the Trisomy 21 Research Society. He serves on committees for the Alzheimer's Project San Diego, the American Neurological Association, and a NIH COBRE Grant to the University of Nebraska. W.C.M. received a royalty payment under a patent held by Stanford University, licensed to Curasen. Other authors declare no competing interests. Author disclosures are available in the Supporting Information.

## CONSENT STATEMENT

All the *post mortem* tissues were donated by individuals or families who have given prior consent for scientific research.

## Supporting information



Supporting Information

Supporting Information
