# Supplementary material for: Tau pathology differs by sex in Alzheimer's disease in Down syndrome
Source: Alzheimers Dement. 2025 Oct 23;21(10):e70838. doi: 10.1002/alz.70838 (PMC12547191; doi:10.1002/alz.70838)
Supplement: Supplementary file 1 — Supporting Information [file ALZ-21-e70838-s001.docx]

**Supplementary Methods**

**Postmortem human sample processing**

The DS-AD group included 18 individuals (ages 41–65), the DS group comprised 10 individuals (ages 22–62), the DS-AD control group comprised 18 individuals (ages 39–59), and the DS control group consisted of 12 individuals (ages 39–59). One previously reported rare case of PT was also included [1]. The AD dementia status of DS-AD cases was confirmed in NIH NeuroBioBank samples. Approximately 10 to 20 mg of frozen tissue was homogenized in 0.5 ml of lysis buffer (50 mM Tris–HCl pH 7.4, 150 mM NaCl, 1% NP-40, 0.5% sodium deoxycholate, 0.1% SDS, and Halt Protease and Phosphatase Inhibitor Cocktail [78440, ThermoFisher Scientific, Waltham, MA]) and rotated at 4°C for 30 minutes. Lysates were centrifuged (15,600 g for 15 minutes at 4°C) to produce supernatants, and the protein concentration was determined using the Bradford assay (5000001; Bio-Rad Laboratories, Hercules, CA). Samples were analyzed by Western blot.

**Sarkosyl fractionation**

Human frontal cortex tissue (Tables S1, S2) was homogenized in ice-cold RIPA buffer as above using a Bullet Blender tissue homogenizer (Next Advance; Troy, NY) at speed 4 for two 1-minute cycles, with cooling intervals on ice to minimize protein degradation. Lysates were centrifuged at 12,000 × g for 10 minutes at 4°C, and the supernatant was collected.

To extract sarkosyl-soluble and -insoluble protein fractions, 1% (w/v) N-lauroylsarcosine (sarkosyl) was added to the supernatant and incubated for 1 hour at room temperature with continuous rotation. Samples were ultracentrifuged at 150,000 × g for 30 minutes at 4°C in a Beckman Optima™ TLX ultracentrifuge (Beckman Coulter, Brea, CA). The supernatant was collected as the sarkosyl-soluble fraction. The pellet was washed with cold PBS and ultracentrifuged again under the same conditions. The final pellet was resuspended in 1× SDS loading buffer, denatured at 95°C for 10 minutes, and used for immunoblotting analysis of the sarkosyl-insoluble fraction.

**SDS-PAGE immunoblotting**

For Western blots, the DS-AD group and the control group were compared, each consisting of 18 samples. We examined portions of samples from each group across three separate runs of SDS-PAGE, followed by Western immunoblottings. This process was applied to both the RIPA and sarkosyl extractions. The data in Figure 1 shows the results from one set of six samples from each group. Gels were quantified using Image Lab (Bio-Rad), and only values within the linear range were used. We normalized all samples relative to actin. The data from all 18 samples in each group were used to calculate the mean, which was then submitted to statistical testing before plotting the results. Each sample was examined only once.

The same procedures were performed for the DS group and its control group. In this case, we had a total of 10 DS samples and 12 controls. However, due to sample shortage, we only examined one DS group of 7 samples and one control group of 6 samples for the sarkosyl extraction (Figure 2H).

Equal amounts of total proteins (10–20 μg per sample) were separated on SDS-PAGE and transferred to PVDF membranes (Bio-Rad). Membranes were blocked with 5% nonfat milk for 1 hour and probed with primary antibodies (mouse antibodies anti-PHF1 tau (1:2,000; a kind gift of Dr. Peter Davies; Albert Einstein University), anti-tau (tau-5; 1:2,000; MA5-12808, ThermoFisher Scientific, Waltham, MA), and anti-β-actin (1:10,000; 60008-1-Ig, Proteintech, Rosemont, IL)) overnight at 4°C. After washing, membranes were incubated with HRP-conjugated goat anti-mouse secondary antibody (1:15,000; 115-035-003, Jackson ImmunoResearch, West Grove, PA) for 1 hour at room temperature. Blots were developed using Bio-Rad Clarity Western ECL substrate and imaged on a ChemiDoc XRS+ system (Bio-Rad). Only signals within the linear range were quantified using ImageLab 3.0.1 software (Bio-Rad). Each dot in the statistical panels represents the value of a sample from an individual case.

**RNA isolation and quantitative PCR**

Total RNA was extracted from human postmortem frontal cortex tissue (Table S3) using the Quick-RNA Miniprep Kit (R1054; Zymo Research, Orange, CA). Equal amounts of RNA were reverse-transcribed into cDNA using the iScript™ cDNA Synthesis Kit (1708891; Bio-Rad), following the manufacturer’s protocol. qPCR was performed for 40 cycles using *MAPT* human primers: forward 5′-ACAGCCACCTTCTCCTCCTC, and reverse 5′-GATCTTCCATCACTTCGAACTCC. Human *ACTB* served as the internal control (forward: 5′-GTCACACTTCATGATGGAGTTGAAGG; reverse: 5′-GACCTGACTGACTACCTCATGAAGAT). Amplification values within the log-linear phase were used for analysis via the ΔΔCt method on an Applied Biosystems 7300 Real-Time PCR System.

**Statistical analysis**

All data are presented as the mean ± SEM. Statistical analyses were performed using PRISM (GraphPad Software, La Jolla, CA) with an ANOVA test followed by the Newman-Keuls multiple comparison test or the Mann-Whitney test. Spearman’s correlation was used to assess a relationship between proteins. The significance levels were **p* < 0.05, ***p* < 0.01, and ****p* < 0.001.

Table S1. Demographics, *post-mortem* interval, and clinical diagnosis for DS-AD, C/DS-AD, and PT-DS frontal cortex samples

| Patient | Gender | Age (Years) | *Post mortem* interval (h) | Clinical history consistent with DS-AD | Neuropathological findings consistent with DS-AD |
| --- | --- | --- | --- | --- | --- |
| C/DS-AD 1* | Female | 49 | 7 | (-) Cognitively normal | NP |
| C/DS-AD 2 | Female | 50 | 7 | (-) Cognitively normal | NP |
| C/DS-AD 3 | Female | 46 | 18 | (-) Cognitively normal | NP |
| C/DS-AD 4* | Male | 48 | 6 | (-) Cognitively normal | (-) |
| C/DS-AD 5* | Male | 57 | 16 | (-) Cognitively normal | NP |
| C/DS-AD 6* | Male | 58 | 19 | (-) Cognitively normal | NP |
| C/DS-AD 7* | Female | 59 | 5.9 | (-) Cognitively normal | (-) |
| C/DS-AD 8* | Female | 39 | 19 | (-) Cognitively normal | NP |
| C/DS-AD 9 | Female | 54 | 6 | (-) Cognitively normal | NP |
| C/DS-AD 10 | Male | 58 | 9 | (-) Cognitively normal | (-) |
| C/DS-AD 11 | Male | 59 | 5.9 | (-) Cognitively normal | (+) Mild amyloid angiopathy |
| C/DS-AD 12 | Male | 59 | 15.8 | (-) Cognitively normal | (-) |
| C/DS-AD 13* | Female | 57 | 7 | (-) Cognitively normal | (-) |
| C/DS-AD 14* | Female | 48 | 8 | (-) Cognitively normal | (-) |
| C/DS-AD 15* | Female | 51 | 21 | (-) Cognitively normal | NP |
| C/DS-AD 16* | Male | 52 | 4 | (-) Cognitively normal | (-) |
| C/DS-AD 17* | Male | 51 | 17 | (-) Cognitively normal | (-) |
| C/DS-AD 18* | Male | 55 | 22 | (-) Cognitively normal | (-) |
| Mean ± SEM | 9F/9M | 52.8 ± 1.3 | 11.9 ± 1.5 |  |  |
|  |  |  |  |  |  |
| DS-AD 1 | Female | 51 | 4 | (+) Dementia | (+) Senile cerebral disease, Alzheimer’s type; congophilic angiopathy |
| DS-AD 2 | Female | 46 | 7 | (+) Continued cognitive delay, declining verbal skill and functional status | (+) Diffuse and severe |
| DS-AD 3 | Female | 57 | 6 | (+) Dementia | (+) Brain atrophy; classic, extensive amyloid deposition and neurofibrillary degeneration |
| DS-AD 4 | Male | 53 | 23 | (+) | (+) AD neuropathological changes and cerebral amyloid angiopathy |
| DS-AD 5 | Male | 55 | 12 | (+) | (+) AD neuropathological changes and cerebral amyloid angiopathy |
| DS-AD 6 | Male | 57 | 5 | (+) | (+) Advanced AD (A3/B3/C3), cerebral amyloid angiopathy |
| DS-AD 7 | Male | 57 | 3 | (+) Alzheimer’s disease with early onset | NP |
| DS-AD 8 | Female | 55 | 25 | (+) | NP |
| DS-AD 9 | Male | 65 | 10 | (+) | (+) Generalized cerebral atrophy, diffuse and severe |
| DS-AD 10 | Male | 41 | 15 | (+) | (+) Advanced, Braak Stage VI, cerebral amyloid angiopathy |
| DS-AD 11 | Male | 56 | 16 | (+) | (+) Advanced |
| DS-AD 12 | Male | 64 | 20 | (+) MMSE 21/30 9 years before death | (+) Minimally atrophic brain with Alzheimer-type neuropathologic changes |
| DS-AD 13 | Male | 53 | 24 | (+) | NP |
| DS-AD 14 | Female | 45 | 2.75 | (+) | (+) Braak Stage VI |
| DS-AD 15# | Female | 47 | 6.5 | (+) | (+) Braak Stage VI |
| DS-AD 16 | Female | 50 | 5 | (+) | (+) Braak Stage VI |
| DS-AD 17 | Female | 52 | 4.37 | (+) | (+) Braak Stage VI |
| DS-AD 18 | Female | 62 | 2.42 | (+) | (+) Braak Stage VI |
| Mean ± SEM | 9F/9M | 53.7 ± 1.5 | 10.6 ± 1.9 |  |  |
|  |  |  |  |  |  |
| PT-DS | Male | 72 | 4.87 | (-) Partial trisomy 21 and cognitively normal | (-) Only a single neuritic plaque and neurofibrillary degeneration consistent with normal aging but not AD |

Abbreviations: C/, control for; DS-AD, Alzheimer’s disease with Down syndrome; PT-DS, Down syndrome with partial trisomy 21; AD, Alzheimer’s disease; F, female; M, male; SEM, standard error of mean. The samples marked with * indicate control samples shared between the DS-AD and DS groups (Tables S1 and S2). The sample marked with # was not processed for sarkosyl fractionation due to insufficient tissue. The samples underlined are represented in the figures as DS-AD and their corresponding CN.

Table S2. Demographics, *post-mortem* interval, and clinical diagnosis for DS and C/DS frontal cortex samples

| Patient | Gender | Age (Years) | *Post-mortem* interval (h) | Clinical history consistent with DS-AD | Neuropathological findings consistent with DS-AD |
| --- | --- | --- | --- | --- | --- |
| C/DS 1 * | Female | 48 | 8 | (-) Cognitively normal | NP |
| C/DS 2 * | Female | 51 | 21 | (-) Cognitively normal | NP |
| C/DS 3 * | Female | 57 | 7 | (-) Cognitively normal | (-) |
| C/DS 4 * | Male | 52 | 4 | (-) Cognitively normal | (-) |
| C/DS 5 * | Male | 51 | 17 | (-) Cognitively normal | (-) |
| C/DS 6 * | Male | 55 | 22 | (-) Cognitively normal | (-) |
| C/DS 7 | Female | 49 | 7 | (-) Cognitively normal | NP |
| C/DS 8 | Female | 39 | 19 | (-) Cognitively normal | NP |
| C/DS 9 | Female | 59 | 5.9 | (-) Cognitively normal | (-) |
| C/DS 10 | Male | 48 | 6 | (-) Cognitively normal | (-) |
| C/DS 11 | Male | 57 | 16 | (-) Cognitively normal | NP |
| C/DS 12 | Male | 58 | 19 | (-) Cognitively normal | (-) |
| Mean ± SEM | 6F/6M | 52.0 ± 1.6 | 12.7 ± 2.0 |  |  |
|  |  |  |  |  |  |
| DS 1 * | Female | 50 | 23 | (-) Trisomy 21 without AD | NP |
| DS 2 * | Female | 39 | 12 | (-) Trisomy 21 without AD | (-) |
| DS 3 * | Male | 22 | 15 | (-) Trisomy 21 without AD | NP |
| DS 4 * | Male | 40 | 10 | (-) Trisomy 21 without AD | Diffuse beta-amyloid deposition in the neocortex |
| DS 5 * | Male | 25 | 22 | (-) Trisomy 21 without AD | Rare neocortical diffuse plaques |
| DS 6 * | Male | 25 | 24 | (-) Trisomy 21 without AD | NP |
| DS 7 * | Male | 23 | 24 | (-) Trisomy 21 without AD | NP |
| DS 8 | Female | 48 | 18.4 | (-) Trisomy 21 without AD | Braak stage III |
| DS 9 | Female | 51 | 2.7 | (-) Trisomy 21 without AD | Braak stage III |
| DS 10 | Female | 62 | 7 | (-) Trisomy 21 without AD | Braak stage III |
| Mean ± SEM | 5F/5M | 38.5 ± 4.5 | 15.8 ± 2.4 |  |  |

Abbreviations: C/, control for; DS, Down syndrome without Alzheimer’s disease; AD, Alzheimer’s disease; F, female; M, male; SEM, standard error of mean. Samples marked with * were used for sarkosyl fractionation. The samples underlined are represented in the figures as DS and their corresponding CN.

Table S3. Demographics, *post-mortem* interval, and clinical diagnoses of frontal cortex samples from DS, C/DS, DS-AD, and C/DS-AD used for RT-PCR analysis

| Patient | Gender | Age (Years) | *Post-mortem* interval (h) | Diagnosis |
| --- | --- | --- | --- | --- |
| control | Female | 48 | 8 | Cognitively normal |
| control | Female | 51 | 21 | Cognitively normal |
| control | Female | 49 | 7 | Cognitively normal |
| control | Female | 59 | 5.9 | Cognitively normal |
| control | Female | 39 | 19 | Cognitively normal |
| control | Female | 50 | 7 | Cognitively normal |
| control | Female | 46 | 18 | Cognitively normal |
| control | Male | 52 | 4 | Cognitively normal |
| control | Male | 57 | 6.7 | Cognitively normal |
| control | Male | 51 | 17 | Cognitively normal |
| control | Male | 59 | 15.8 | Cognitively normal |
| control | Male | 59 | 5.9 | Cognitively normal |
| control | Male | 48 | 6 | Cognitively normal |
| control | Male | 58 | 19 | Cognitively normal |
| control | Male | 57 | 16 | Cognitively normal |
|  |  |  |  |  |
| DS-AD | Female | 57 | 6 | Trisomy 21 with AD |
| DS-AD | Female | 55 | 25 | Trisomy 21 with AD |
| DS-AD | Female | 46 | 7 | Trisomy 21 with AD |
| DS-AD | Female | 45 | 2.75 | Trisomy 21 with AD |
| DS-AD | Female | 62 | 2.42 | Trisomy 21 with AD |
| DS-AD | Female | 52 | 4.37 | Trisomy 21 with AD |
| DS-AD | Male | 53 | 24 | Trisomy 21 with AD |
| DS-AD | Male | 55 | 12 | Trisomy 21 with AD |
| DS-AD | Male | 57 | 5 | Trisomy 21 with AD |
| DS-AD | Male | 64 | 20 | Trisomy 21 with AD |
| DS-AD | Male | 57 | 3 | Trisomy 21 with AD |
| DS-AD | Male | 41 | 15 | Trisomy 21 with AD |
| DS-AD | Male | 56 | 16 | Trisomy 21 with AD |
|  |  |  |  |  |
| DS | Female | 50 | 23 | Trisomy 21 without AD |
| DS | Female | 39 | 12 | Trisomy 21 without AD |
| DS | Female | 42 | 5 | Trisomy 21 without AD |
| DS | Female | 48 | 18.4 | Trisomy 21 without AD |
| DS | Female | 51 | 2.7 | Trisomy 21 without AD |
| DS | Female | 62 | 7 | Trisomy 21 without AD |
| DS | Male | 22 | 15 | Trisomy 21 without AD |
| DS | Male | 40 | 10 | Trisomy 21 without AD |
| DS | Male | 25 | 22 | Trisomy 21 without AD |
| DS | Male | 25 | 24 | Trisomy 21 without AD |
| DS | Male | 23 | 24 | Trisomy 21 without AD |
| DS | Male | 57 | 22 | Trisomy 21 without AD |

Abbreviations: DS-AD, Alzheimer’s disease with Down syndrome; DS, Down syndrome without Alzheimer’s disease; AD, Alzheimer’s disease.

Table S4. Analysis of PMI and age between sexes in DS-AD and control samples (Mann-Whitney test).

| Sample comparison | |  | Significance (*P*) |
| --- | --- | --- | --- |
| C/DS-AD/F | C/DS-AD/M | PMI | 0.8942 |
| DS-AD/F | DS-AD/M | PMI | 0.0575 |
| C/DS-AD/F | DS-AD/M | PMI | 0.4258 |
| DS-AD/F | C/DS-AD/M | PMI | 0.0771 |
|  |  |  |  |
| C/DS/F | C/DS/M | PMI | 0.8723 |
| DS/F | DS/M | PMI | 0.2222 |
| C/DS/F | DS/M | PMI | 0.0547 |
| DS/F | C/DS/M | PMI | 0.9307 |
|  | | | |
| C/DS-AD/F | C/DS-AD/M | Age | 0.0693 |
| DS-AD/F | DS-AD/M | Age | 0.1213 |
| C/DS-AD/F | DS-AD/M | Age | 0.0927 |
| DS-AD/F | C/DS-AD/M | Age | 0.1109 |
|  |  |  |  |
| C/DS/F | C/DS/M | Age | 0.5196 |
| DS/F | DS/M | Age | 0.0159* |
| C/DS/F | DS/M | Age | 0.0087** |
| DS/F | C/DS/M | Age | 0.3131 |

Abbreviations: C/, control for; DS-AD, Alzheimer’s disease with Down syndrome; F, female; M, male

Table S5. Spearman’s correlation analysis between PMI or age and total tau or PHF1 tau in either control, DS-AD or DS samples

| Sample type |  |  | Sample size (*n*) | Correlation coefficient (*r*) | Significance (*P*) |
| --- | --- | --- | --- | --- | --- |
| C/DS-AD/F  whole lysate | PMI | Total tau | 9 | -0.4238 | 0.2499 |
| C/DS-AD/M  whole lysate | PMI | Total tau | 9 | -0.4000 | 0.2912 |
| DS-AD/F  whole lysate | PMI | Total tau | 9 | -0.3000 | 0.4366 |
| DS-AD/M  whole lysate | PMI | Total tau | 9 | -0.03333 | 0.9484 |
| DS-AD/F  whole lysate | PMI | PHF1 tau | 9 | -0.2500 | 0.5206 |
| DS-AD/M  whole lysate | PMI | PHF1 tau | 9 | 0.0000 | 1.0184 |
| DS-AD/F  sarkosyl-insoluble | PMI | Total tau | 8 | -0.4048 | 0.3268 |
| DS-AD/M  sarkosyl-insoluble | PMI | Total tau | 9 | 0.01667 | 0.9816 |
| DS-AD/F  sarkosyl-insoluble | PMI | PHF1 tau | 8 | -0.5952 | 0.1323 |
| DS-AD/M  sarkosyl-insoluble | PMI | PHF1 tau | 9 | 0.0000 | 1.0184 |
| C/DS-AD/F  sarkosyl-soluble | PMI | Total tau | 9 | -0.3221 | 0.3853 |
| C/DS-AD/M  sarkosyl-soluble | PMI | Total tau | 9 | -0.2333 | 0.5517 |
| DS-AD/F  sarkosyl-soluble | PMI | Total tau | 8 | -0.6429 | 0.0962 |
| DS-AD/M  sarkosyl-soluble | PMI | Total tau | 9# | -0.7333 | 0.0311* |
|  |  |  |  |  |  |
| C/DS/F  whole lysate | PMI | Total tau | 6 | 0.2319 | 0.6583 |
| C/DS/M  whole lysate | PMI | Total tau | 6 | 0.08571 | 0.9194 |
| DS/F  whole lysate | PMI | Total tau | 5 | 0.5000 | 0.4500 |
| DS/M  whole lysate | PMI | Total tau | 5 | -0.8208 | 0.1333 |
| C/DS/F  sarkosyl-soluble | PMI | Total tau | 3 | 1.000 | 0.3333 |
| C/DS/M  sarkosyl-soluble | PMI | Total tau | 3 | 0.5000 | 1.0000 |
| DS/F  sarkosyl-soluble | PMI | Total tau | 2 | NA | NA |
| DS/M  sarkosyl-soluble | PMI | Total tau | 5 | 0.05130 | 0.9500 |
|  |  |  |  |  |  |
| C/DS-AD/F  whole lysate | age | Total tau | 9 | 0.7167 | 0.0369* |
| C/DS-AD/M  whole lysate | age | Total tau | 9 | -0.1933 | 0.6134 |
| DS-AD/F  whole lysate | age | Total tau | 9 | 0.01667 | 0.9816 |
| DS-AD/M  whole lysate | age | Total tau | 9 | -0.4538 | 0.2298 |
| DS-AD/F  whole lysate | age | PHF1 tau | 9 | 0.03333 | 0.9484 |
| DS-AD/M  whole lysate | age | PHF1 tau | 9 | -0.06723 | 0.8801 |
| DS-AD/F  sarkosyl-insoluble | age | Total tau | 8 | 0.5952 | 0.1323 |
| DS-AD/M  sarkosyl-insoluble | age | Total tau | 9 | 0.3025 | 0.4366 |
| DS-AD/F  sarkosyl-insoluble | age | PHF1 tau | 8 | 0.5476 | 0.1710 |
| DS-AD/M  sarkosyl-insoluble | age | PHF1 tau | 9 | 0.1092 | 0.7756 |
| C/DS-AD/F  sarkosyl-soluble | age | Total tau | 9 | 0.8167 | 0.0108* |
| C/DS-AD/M  sarkosyl-soluble | age | Total tau | 9 | 0.06723 | 0.8801 |
| DS-AD/F  sarkosyl-soluble | age | Total tau | 8 | -0.3333 | 0.4279 |
| DS-AD/M  sarkosyl-soluble | age | Total tau | 9 | 0.2605 | 0.4933 |
|  |  |  |  |  |  |
| C/DS/F  whole lysate | age | Total tau | 6 | 0.3714 | 0.4972 |
| C/DS/M  whole lysate | age | Total tau | 6 | 0.4857 | 0.3556 |
| DS/F  whole lysate | age | Total tau | 5 | -0.5000 | 0.4500 |
| DS/M  whole lysate | age | Total tau | 5 | 0.3591 | 0.5167 |
| C/DS/F  sarkosyl-soluble | age | Total tau | 3 | -0.5000 | 1.0000 |
| C/DS/M  sarkosyl-soluble | age | Total tau | 3 | 1.000 | 0.3333 |
| DS/F  sarkosyl-soluble | age | Total tau | 2 | NA | NA |
| DS/M  sarkosyl-soluble | age | Total tau | 5 | -0.6669 | 0.2333 |

Abbreviations: DS-AD, Alzheimer’s disease with Down syndrome; F, female; M, male; NA, not available; #, Studentized residuals and Cook’s distance tests for regression outliers did not identify any strong outliers.

Figure S1. Western blot images for Figure 1.
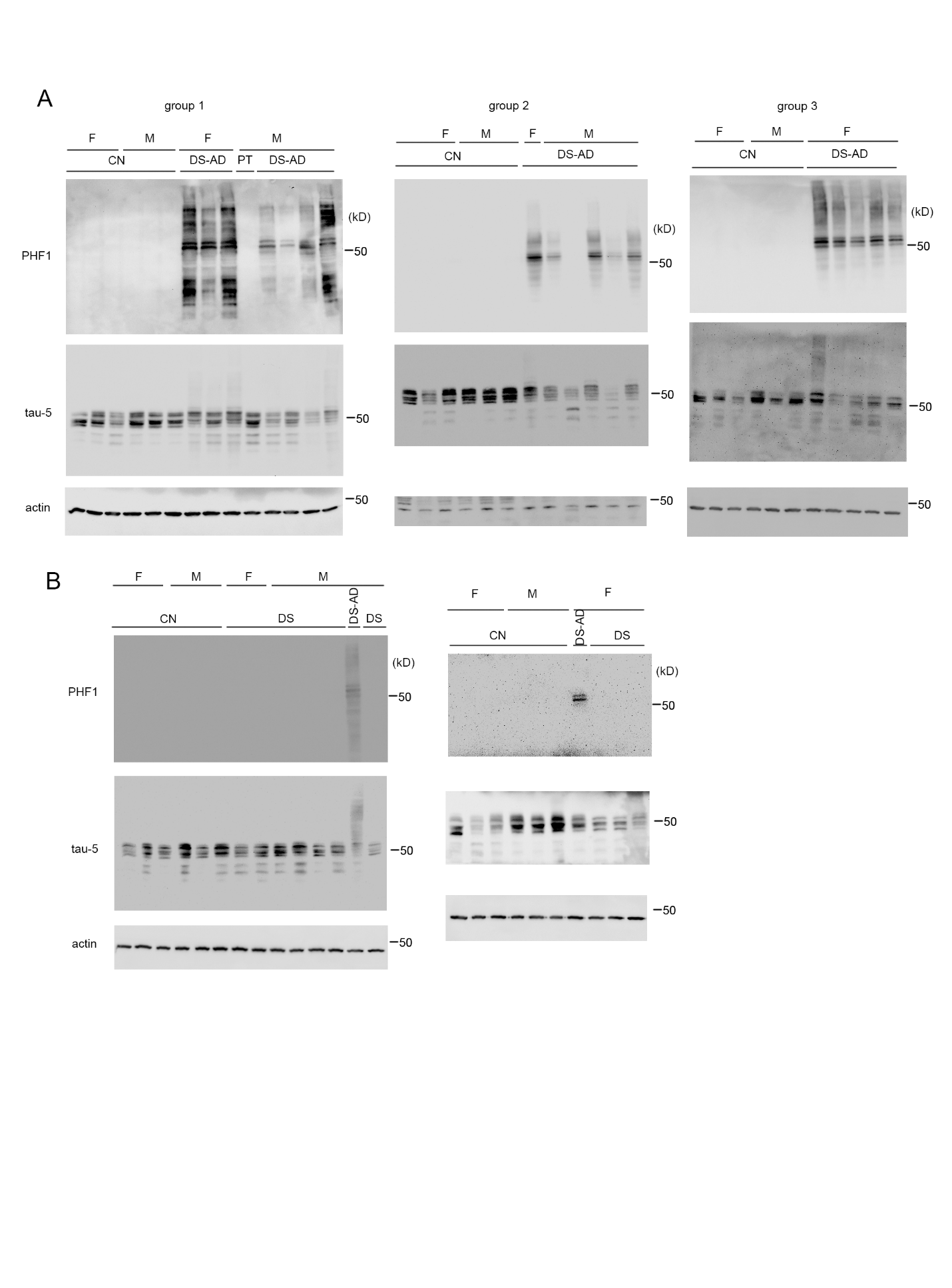


Figure S2. Western blot images for Figure 2.
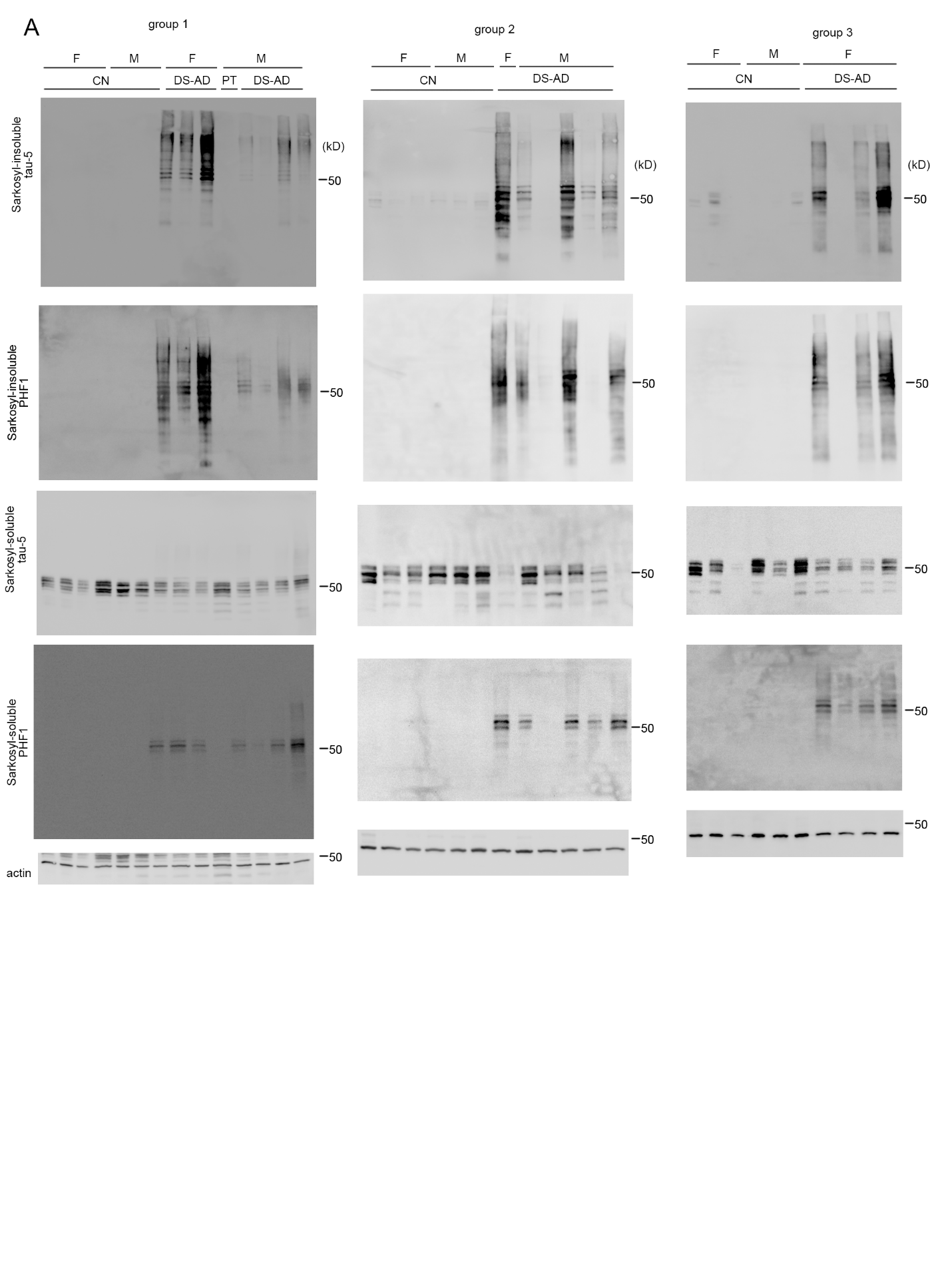


Reference

[1] Doran E, Keator D, Head E, Phelan MJ, Kim R, Totoiu M, et al. Down Syndrome, Partial Trisomy 21, and Absence of Alzheimer's Disease: The Role of APP. J Alzheimers Dis. 2017;56:459-70.
